# Moderating Effects of Striving to Avoid Inferiority on Income and Mental Health

**DOI:** 10.3389/fpsyg.2022.838991

**Published:** 2022-06-03

**Authors:** Asa Nagae, Kenichi Asano, Yasuhiro Kotera

**Affiliations:** ^1^Graduate School of Psychology, Mejiro University, Tokyo, Japan; ^2^National Center for Cognitive Behavior Therapy and Research, National Center of Neurology and Psychiatry, Tokyo, Japan; ^3^School of Health Sciences, University of Nottingham, Nottingham, United Kingdom

**Keywords:** inferiority, mental health – related quality of life, well-being, income, social safeness, social comparison

## Abstract

Many people experience feelings of inferiority in their life. The concept of striving to avoid inferiority is a belief associated with the unwanted fear of being overlooked, missing out on opportunities for advancement, and active rejection. This study examined the effect of striving to avoid inferiority on mental health and well-being. We hypothesized that striving to avoid inferiority would modify the relationship among socioeconomic status, mental health, and well-being, therefore examined the effect of striving to avoid inferiority on the relationship between annual income, mental health, and well-being. The participants were 491 adults (241 men and 250 women). The results indicated that insecure striving (IS) in competition with others positively correlated with depression, anxiety, and stress, whereas secure non-striving (SNS) in acceptance of inferiority positively correlated with the satisfaction with life and negatively correlated with depression. The effect of striving to avoid inferiority on the relationship among annual income, mental health, and well-being indicated that SNS affected the relationships between annual income and well-being, annual income and depression, income and anxiety, and the interaction between IS and SNS. Moreover, the relationship between income and stress was influenced by SNS and the interaction between IS and SNS. These results indicated that SNS or the interaction between IS and SNS were the only variables regulating the relationship among annual income, mental health, and well-being. These findings suggested that accepting feelings of inferiority or striving to avoid inferiority influences the mental health and well-being of people.

## Introduction

Gilbert (1989, 2005a,b), and Dykman (1998) stated that people who considered themselves insecure or unsafe in social relationships were more inclined to feel inferior. This is related to the psychological pressure forcing them to make efforts to avoid undesirable situations, such as “not being noticed” and “being rejected.” Such tendencies are often discussed in the contexts of perfectionism, and many studies revealed their relations to mental health problems (Dunkley and Blankstein, 2000; Dunkley et al., 2003, 2006, 2009). These reports underpin the critical roles of fear of rejection or being overlooked and involuntary effort to avoid inferiority.

The belief that one must strive to avoid inferiority is defined as “striving to avoid inferiority” (Gilbert et al., 2007). The concept of striving to avoid inferiority is a belief associated with the fear of being overlooked, missing out on opportunities for advancement, and active rejection. It has been observed to predict depression and anxiety more than inferiority (Gilbert et al., 2007). Therefore, individuals with a high level of striving to avoid inferiority view others as rejecting and shaming. In addition, even in a situation in which an individual is aware that he or she is not inferior to others, the pressure and fear of past inferiority may activate the striving to avoid inferiority, which may threaten mental health. Oppositely, Gilbert (1989, 2005a,b), and Dykman (1998) stated that there are a certain number of people who regard themselves as accepting and helpful, do not fear inferiority or mistakes, and feel safe in social relationships.

Here, the striving to avoid inferiority has been described as a two-factor structure, called insecure striving (IS) and secure non-striving (SNS; Gilbert et al., 2007). IS is associated with being overlooked, missing out, and active rejection. Ferreira et al. (2013) observed that IS was associated with low social status and low self-esteem among women. Ferreira et al. (2013) argued that IS had a significant moderating effect on low social status and influenced thinness and dieting behaviors among women, supporting the hypothesis that the desire to lose weight arises as a competitive weapon to secure social status. In fact, a more recent study revealed IS can be a key moderator between shame and disordered eating (Ferreira and Mendes, 2020). Gilbert et al. (2007), who proposed this concept, observed that IS was associated with depression, anxiety, and stress in depressed populations, as well as feelings of inferiority, beliefs that others do not particularly value them, and beliefs that they were being looked down upon. The relationship between IS and distress is replicated in undergraduate student and athlete samples (Walton et al., 2020; To et al., 2021). Other survey studies identified that IS was correlated with daily hassles and paranoia positively, and with self-mastery negatively (Anderson and Freeman, 2013). A recent review concluded that upward mobility can be a risk for lower health state because of its competitive environment and effortful strivings (Chen et al., 2022), also suggesting IS relates to psychopathological symptoms.

Conversely, SNS is associated with feelings of acceptance of oneself and by others, regardless of success or failure, whether one has accomplished something or not (Bellew et al., 2006). Therefore, there was no need to address SNS as acutely as IS was addressed. SNS comprises feelings that focus on the sense of being accepted by others, such as “If I make mistakes‚ I know other people will still like me” and “I do not have to prove myself to feel part of a group.” Thus, SNS is a factor behind the sense of security of being socially accepted by others. The aforementioned study revealed SNS correlates to daily hassles and paranoia negatively (Anderson and Freeman, 2013).

In addition, SNS had a significant negative relationship with variables associated with IS (Gilbert et al., 2009). Bellew et al. (2006) discovered that SNS was associated with few depressive symptoms, less anxiety concerning appearance, less negative eating habits, and less anxiety concerning rejection. In addition, they observed that feelings of security were associated with making favorable social comparisons.

One of the domains and socioeconomic topics about which people might feel inferior is income. Certain reports have shown that low socioeconomic status is associated with poor mental health (Lorant et al., 2003). Kahneman and Deaton (2010) divided happiness into the following two types: emotional and long-term happiness, and observed that emotional happiness correlated with income and feelings of “joy” and “satisfaction” for those whose annual income was less than $75,000. However, when the annual income exceeded $75,000, the relationship between income and feelings of joy and satisfaction weakened. Frank (2013) stated that income is a status for which satisfaction can be obtained, compared with those of others. Therefore, the association between income and happiness or satisfaction can be influenced in comparison with the income of other people. It was reported that the incidence of mental illness and crime is higher in competitive societies than that in caring societies (Arrindell et al., 2003). Thus, it is possible that the relationship between “socioeconomic status” and “happiness and mental health” is influenced by the striving to avoid inferiority. Anderson and Freeman (Anderson and Freeman, 2013) investigated a relationship between socioeconomic status and IS indirectly by using money-related hassles, and found a significant correlation between them. Another report also showed a significant relation between ill-being and socioeconomic status (Headey et al., 1984). However, the relationship among socioeconomic status, happiness or well-being, and the striving to avoid inferiority has not been enough clarified.

The purpose of this study was to examine the relationship between striving to avoid inferiority and mental health and well-being. In addition, hypothesizing that the striving to avoid inferiority is a factor that adjusts the relationship between socioeconomic status, mental health and well-being, the purpose of this study was to examine the effect of the striving to avoid inferiority on the relationship among annual income, mental health, and well-being.

## Materials and Methods

### Procedure

We conducted an anonymous, individual-response, internet-based survey of the general adult (over 20 years old) population in Japan in November 2020, using the panel survey service by Questant. We received 547 responses. Of these, 491 (241 males, 250 females; mean age, 45.01 years, SD, 13.99) were valid respondents, excluding those with incomplete survey items.

In the online questionnaire, it was stated that participation in the survey was not compulsory, that there would be no disadvantage if respondents refused to answer, the results of the survey would be used only for research purposes, and individual responses would not be published in their entirety. The results of the survey will be used for research purposes only, and individual responses will not be publicized. This study was approved by the Research Ethics Committee of Mejiro University in the Humanities and Social Sciences (Approval No. 20 person-014).

### Questionnaire

We asked questions regarding gender, age, marital status, and employment status. The respondents were asked to choose from the following options: full-time (approximately 40 h per week), part-time (a few hours per week), self-employed (irregular/unfixed working hours), retired, housewife (husband), student, other, and unanswered.

Household income, which is a socioeconomic condition, was used to measure income in Japanese yen. Household income is defined as the annual income of the entire household, including the face value before taxes and insurance premiums, as well as extra and supplementary income.

The Striving to Avoid Inferiority Scale (SAIS) developed by Gilbert et al. (2007) was used. The SAIS was translated into Japanese by two psychologists (KA and YK), and a meeting was held to integrate the translations. The integrated translation was backtranslated by a Japanese–English translation company specializing in psychology. The two backtranslations were sent to the scale developer and were confirmed to be equivalent to the original version. The SAIS comprised two scales, Parts 1 and 2, but only Part 1 was used which was already verified its factor structure. This scale comprised two factors: IS (e.g., I need to seek success to be valued by others) and SNS (e.g., people around me like me even if I make mistakes). A total of 31 items were measured (Table 1). Five items (0, never; 1, almost never; 2, sometimes; 3, almost always; and 4, always) were used.

**Table 1:** Demographic data of the target population.

*Gender*			
	Male (%)	241	(49.08%)
	Female (%)	250	(50.92%)
*Age*			
	Mean (*SD*)	45.01	(13.99)
	Median	45	
	Range	21–69	
*Presence or absence of a spouse*			
	No spouse (never married) (%)	182	(37.06%)
	Bereavement/separation (divorce) (%)	39	(7.94%)
	With spouse (married) (%)	269	(54.79%)
	Unanswered (%)	1	(0.20%)
*Type of work*			
	Full-time (total of about 40 h per week)	219	(44.60%)
	Short-time (part-time, etc.) (a few days a week)	70	(14.26%)
	Self-employed (irregular/fixed working hours)	24	(4.89%)
	After retirement	26	(5.30%)
	Full-time housewife (husband)	92	(18.74%)
	Student	16	(3.26%)
	Other	38	(7.74%)
	Unanswered	6	(1.22%)

To measure mental health, we used the DASS-15 scale (Depression Anxiety Stress Scales-15) developed by Adachi et al. (2013), which is the Japanese version of the DASS 21-item version developed by Lovibond and Lovibond (1995). This scale comprised three factors with 5 items on each scale, namely depression, anxiety, and stressA four-point scale (0, not at all; 1, sometimes; 2, quite often; and 3, very often) was used.

To measure subjective well-being, we used the Satisfaction With Life Scale (SWLS) developed by Sumino (Sumino, 1994), which is a Japanese version of the SWLS developed by Diener et al. (1985). This scale comprised five items responded on a 7-point scale [1 (not at all) to 7 (totally)].

### Analysis

After confirming the reliability of the scales, the mean, standard deviation, minimum, and maximum values of the scores were calculated. Furthermore, a correlation analysis between each variable was conducted. In addition, to examine if the SAIS subscales and household income influenced changes in the SWLS and DASS subscales, we used the SWLS or DASS subscales of depression, anxiety, and stress as the objective variables, and we used the household income, SAIS subscales of IS and SNS, and interaction between household income and IS as the explanatory variables. Hierarchical multiple regression analyses were conducted with the following explanatory variables: the interaction between household income and IS; household income and SNS; and household income, IS, and SNS. In addition, a simple slope analysis was conducted to clarify the effects of the interactions.

Descriptive statistics, correlation analysis, hierarchical multiple regression analysis, and simple slope analysis were conducted using HAD ver. 16.3, a free software (Shimizu, 2016).

## Results

### Demographic Data

The demographic data are shown in Table 1. A total of 241 (49.08%) participants were male and 250 (50.92%) were female. The mean age was 45.01 (SD = 13.99), and the median age was 45, with a range of 21–69 years. A total of 182 (37.06%) participants had no spouse (never married), 39 (7.94%) were bereaved or separated (divorced), 269 (54.79%) had a spouse (married), and one (0.20%) did not answer. Regarding employment status, 219 (44.60%) were full-time workers, 70 (14.26%) were part-time workers, 24 (4.89%) were self-employed, 26 (5.30%) were retired, 92 (18.74%) were full-time housewives (husbands), 16 (3.26%) were students, 38 (7.74%) were others, and 6 (1.22%) did not answer.

The descriptive statistics were calculated and are shown in Table 2.

**Table 2:** Descriptive statistics.

	Mean	SD	Min	Max	α
Household income (yen)	601.19	529.21	0	8500	
SAIS					
IS	51.01	12.08	19	94	0.94
SNS	36.69	7.93	12	60	0.91
DASS-15					
Depression	8.91	3.78	5	20	0.91
Anxiety	7.49	3.34	5	20	0.91
Stress	8.61	3.45	5	20	0.88
SWLS	17.59	6.79	5	35	0.93

SAIS, Striving to avoid Inferiority scale; IS, Insecure strivings; SNS, Secure non-strivings; DASS-15, Depression, anxiety, and stress scale-15; SWLS, Satisfaction with life scale.

The mean household income was 601.19 million yen (SD = 529.21), the minimum was 0 million yen, and the maximum was 85 million yen.

The scores for each subscale were summed, and the scores for each subfactor were calculated. The mean score of “IS” was 51.01 (SD = 12.08), minimum was 19, maximum was 94, and alpha coefficient was alpha = 0.94. The mean score for “SNS” was 36.69 (SD = 7.93), minimum was 12, maximum was 60, and alpha coefficient was alpha = 0.91.

The scores for each factor were calculated by summing the scores for each subscale. The mean score of “depression” was 8.91 (SD = 3.78), minimum was 5, maximum was 20, and alpha coefficient was alpha = 0.91. The mean score for “anxiety” was 7.49 (SD = 3.34), with a minimum of 5, maximum of 20, and alpha coefficient of alpha = 0.91. The mean score for “stress” was 8.61 (SD = 3.45), with a minimum of 5, maximum of 20, and alpha coefficient of alpha = 0.88.

The wellbeing scores were calculated by summing the scores of all five items. The mean score was 17.59 (SD = 6.79), minimum was 5, maximum was 35, and alpha coefficient was alpha = 0.93.

### Correlation Analysis

A correlation analysis was conducted to examine the relationship between striving to avoid inferiority and mental health, well-being, and household income. The results are shown in Table 3.

**Table 3:** Correlation analysis.

S. No.		1	2	3	4	5	6
1.	IS	ー					
2.	SNS	−0.27^**^	ー				
3.	SWLS	−0.08	0.40^**^	ー			
4.	Depression	0.34^**^	−0.33^**^	−0.38^**^	ー		
5.	Anxiety	0.32^**^	−0.24^**^	−0.11^*^	0.74^**^	ー	
6.	Stress	0.35^**^	−0.27^**^	−0.22^**^	0.75^**^	0.82^**^	ー
7.	Household income	−0.02	−0.08	0.20^**^	−0.16^**^	−0.13^**^	−0.17^**^

IS, Insecure strivings; SNS, Secure Non-strivings; SWLS, Satisfaction with life scale.

* *p* < 0.05;

** *p* < 0.01.

The correlation analysis showed that IS had a significant positive correlation (*r* = 0.34, *p* = 0.01) with “depression”; significant positive correlation (*r* = 0.32, *p* < 0.01) with “anxiety”; and significant positive correlation (*r* = 0.35, *p* < 0.01) with “stress.”

SNS was shown to have a significant positive correlation (*r* = 0.40, *p* < 0.01) with “SWLS” and a significant negative correlation (*r* = −0.33, *p* < 0.01) with “depression”; significant negative correlation (*r* = −0.24, *p* < 0.01) with “anxiety”; and significant negative correlation (*r* = −0.27, *p* < 0.01) with “stress.”

Moderating Effects of Striving to Avoid Inferiority on Household Income and Wellbeing.

A hierarchical multiple regression analysis was conducted to examine the effects of the SAIS subscales and household income on changes in SWLS.

The procedure of the hierarchical multiple regression analysis with SWLS as the objective variable was as follows: in Step 1, the SAIS subscales, IS, SNS, and household income were entered into the regression equation. In Step 2, we added the interaction terms of IS and SNS, IS and household income, and SNS and household income to the variables in Step 1. In Step 3, the interaction terms of IS, SNS, and household income were added to the variables in Steps 1 and 2 (Table 4).

**Table 4:** Hierarchical multiple regression analysis with SWLS as the objective variable.

	*R* ^2^	Δ*R*^2^	Adj.*R*^2^	*β*
Step 1	0.18^**^		0.18	
IS				0.03
SNS				0.39^**^
Household income				
Step2	0.19^**^	0.01	0.18	
IS				0.02
SNS				0.38^**^
Household income				
IS × SNS				0.01
IS × Household income				−0.05
SNS × Household income				−0.13^*^
Step3	0.19^**^	0.00	0.18	
IS				0.02
SNS				0.38^**^
Household income				0.23^**^
IS × SNS				0.02
IS × Household income				−0.05
SNS × Household income				−0.13^*^
IS × SNS × Household income				0.01

IS, Insecure strivings; SNS, Secure non-strivings.

* *p* < 0.05;

** *p* < 0.01.

The results of the hierarchical multiple regression analysis with SWLS as the objective variable are shown in Table 5. The results showed that the interaction terms of SNS, household income, and SNS and household income were positively associated with SWLS. The increase in *R*^2^ from Steps 1 to 2 and Steps 2 to 3 was 0.01 (*F* = 1.92, *p* < 0.01) and 0.00 (*F* = 0.05, *p* < 0.01), respectively. Cohen’s (Cohen, 1992) effect estimates for small, medium, and large increases in *R*^2^ were 0.02, 0.13, and 0.26, respectively; however, the variance explanatory rate of the effect of the interaction term on the target variable was generally between 1 and 3% (Champoux and Peters, 1987), suggesting that even 1% can be interpreted as a significant result (Fairchild and McQuillin, 2010).

**Table 5:** Hierarchical multiple regression analysis with depression as the objective variable.

	*R* ^2^	Δ*R*^2^	Adj.*R*^2^	*β*
Step 1	0.19^**^		0.19	
IS				0.27^**^
SNS				−0.25^**^
Household income				−0.13^**^
Step2	0.21^**^	0.02^**^	0.20	
IS				0.29^**^
SNS				−0.22^**^
Household income				−0.21^**^
IS × SNS				−0.02
IS × Household income				−0.09^*^
SNS × Household income				−0.19^**^
Step3	0.21^**^	0.00	0.20	
IS				0.28^**^
SNS				−0.22^**^
Household income				−0.19^**^
IS × SNS				0.01
IS × Household income				0.08^*^
SNS × Household income				0.20^**^
IS × SNS × Household income				0.07

IS, Insecure strivings; SNS, Secure non-strivings

* *p* < 0.05;

** *p* < 0.01.

**Figure 1:**
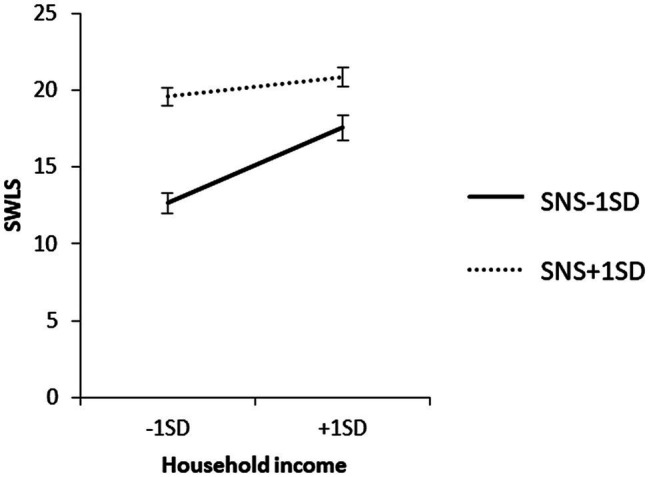
Simple effects analysis of the interaction term SNS × household income for SWLS purposes.

To examine the content of the significant interaction, we conducted a simple slope analysis to examine the effect of household income on each value of the SNS mean ± 1 SD. The results are shown in Figure 1. The results showed that when SNS was high, household income did not have a significant effect on SWLS (*β* = 0.09, nonsignificant (ns)). However, when SNS was low, household income had a significant effect on SWLS (*β* = 0.36, *p* < 0.01).

### Moderating Effects of Striving to Avoid Inferiority on Household Income and Mental Health

Another hierarchical multiple regression analysis was conducted to examine the effects of the SAIS subscales and household income on changes in mental health.

The procedure of the hierarchical multiple regression analysis with depression as the objective variable was as follows: in Step 1, IS, SNS, and household income, which are subscales of the SAIS, were entered into the regression equation. In Step 2, we added the interaction terms of IS and SNS, IS and household income, and SNS and household income to the variables in Step 1. In Step 3, the interaction terms of IS, SNS, and household income were added to the variables in Steps 1 and 2.

The results of the hierarchical multiple regression analysis with depression as the objective variable are shown in Table 5, where the interaction terms of IS, SNS, and household income positively associated with depression, and SNS and household income were negatively associated with depression. The increase in *R*^2^ from Steps 1 to 2 and Steps 2 to 3 was 0.02 (*F* = 3.94, *p* < 0.01) and 0.00 (*F* = 1.65, *p* < 0.01), respectively.

**Figure 2:**
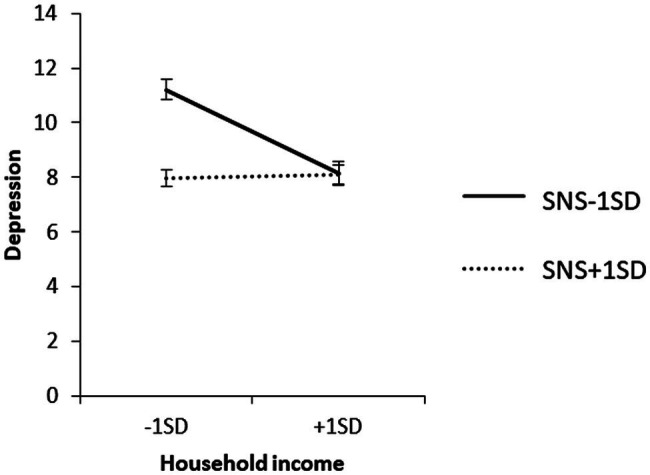
Simple effect analysis of the interaction term SNS × household income for depression.

To examine the content of the significant interaction, the effect of household income on each value of the SNS mean ± 1 SD was confirmed by a simple slope analysis. The results are shown in Figure 2.

As observed from the SNS mean + 1 SD, household income did not have a significant effect on depression when SNS was high (*β* = −0.02, ns).

For the SNS mean − 1 SD, household income had a significant negative effect on depression when SNS was low (*β* = −0.41, *p* < 0.01).

The procedure of the hierarchical multiple regression analysis with anxiety as the objective variable was as follows: In Step 1, the subscales of the SAIS, IS, SNS, and household income were entered into the regression equation. In Step 2, the interaction terms of IS and SNS, IS and household income, and SNS and household income were added to the variables in Step 1. In Step 3, the interaction terms of IS, SNS, and household income were added to the variables in Steps 1 and 2.

The results of the hierarchical multiple regression analysis with anxiety as the objective variable are shown in Table 6, where the interaction terms of IS, SNS, and household income were positively associated with anxiety, while SNS and household income were negatively associated with anxiety. The increase in *R*^2^ from Steps 1 to 2 and Steps 2 to 3 was 0.02 (*F* = 2.99, *p* < 0.01) and 0.01 (*F* = 9.37, *p* < 0.01), respectively.

**Table 6:** Hierarchical multiple regression analysis with anxiety as the objective variable.

	*R* ^2^	Δ*R*^2^	Adj.*R*^2^	*β*
Step 1	0.14^**^		0.13	
IS				0.28^**^
SNS				−0.15^**^
Household income				−0.13^*^
Step2	0.16^**^	0.02^*^	0.15	
IS				0.29^**^
SNS				−0.11^*^
Household income				−0.18^**^
IS × SNS				−0.05
IS × Household income				−0.05
SNS × Household income				−0.15^**^
Step3	0.17^**^	0.02^**^	0.16	
IS				0.28^**^
SNS				−0.10^*^
Household income				−0.14^**^
IS × SNS				0.13^*^
IS × Household income				0.03
SNS × Household income				0.19^**^
IS × SNS × Household income				0.17^**^

IS, Insecure strivings; SNS, Secure non-strivings.

* *p* < 0.05;

** *p* < 0.01.

**Figure 3:**
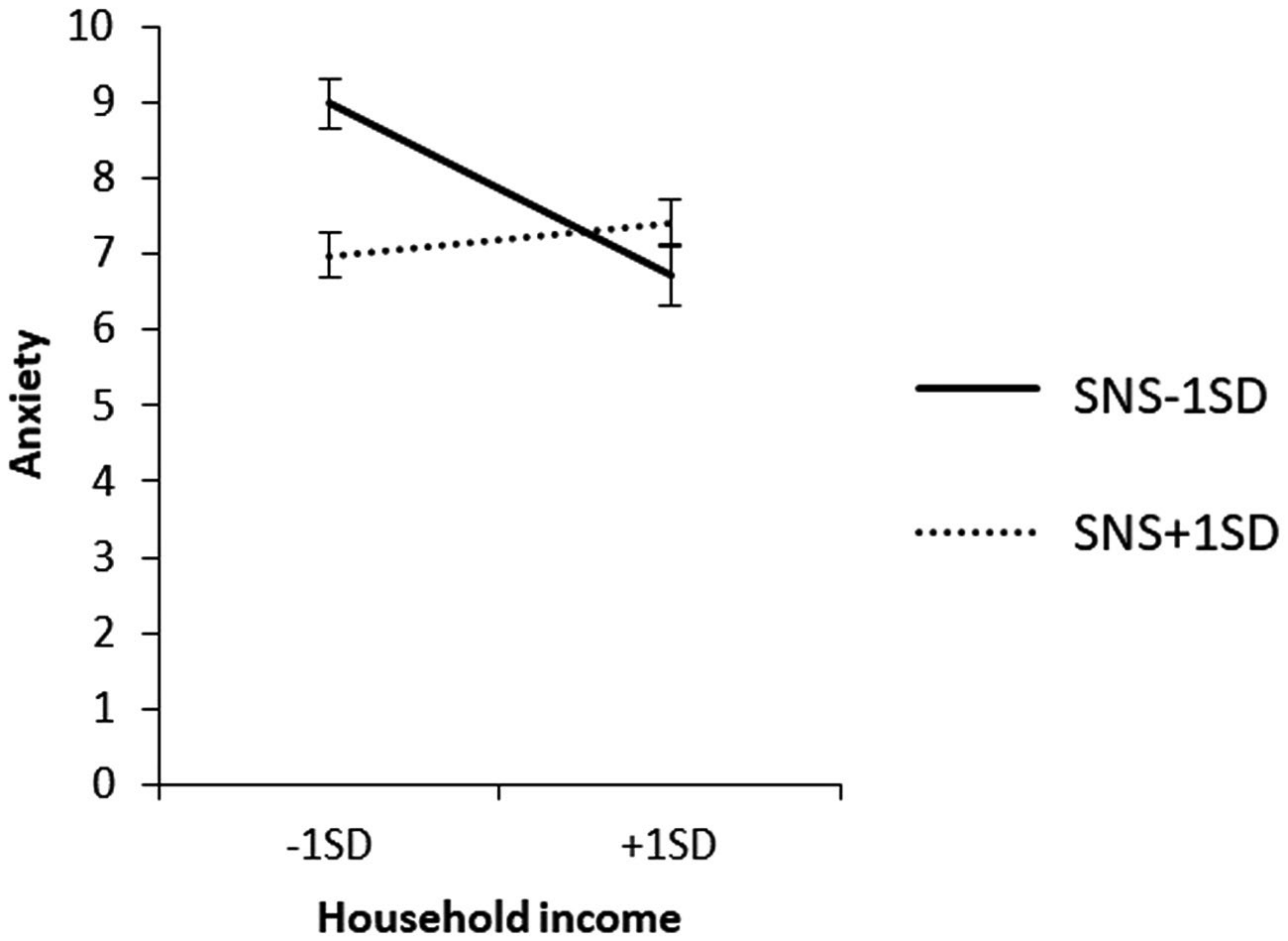
Simple effects analysis of the interaction term SNS × household income for anxiety.

**Figure 4:**
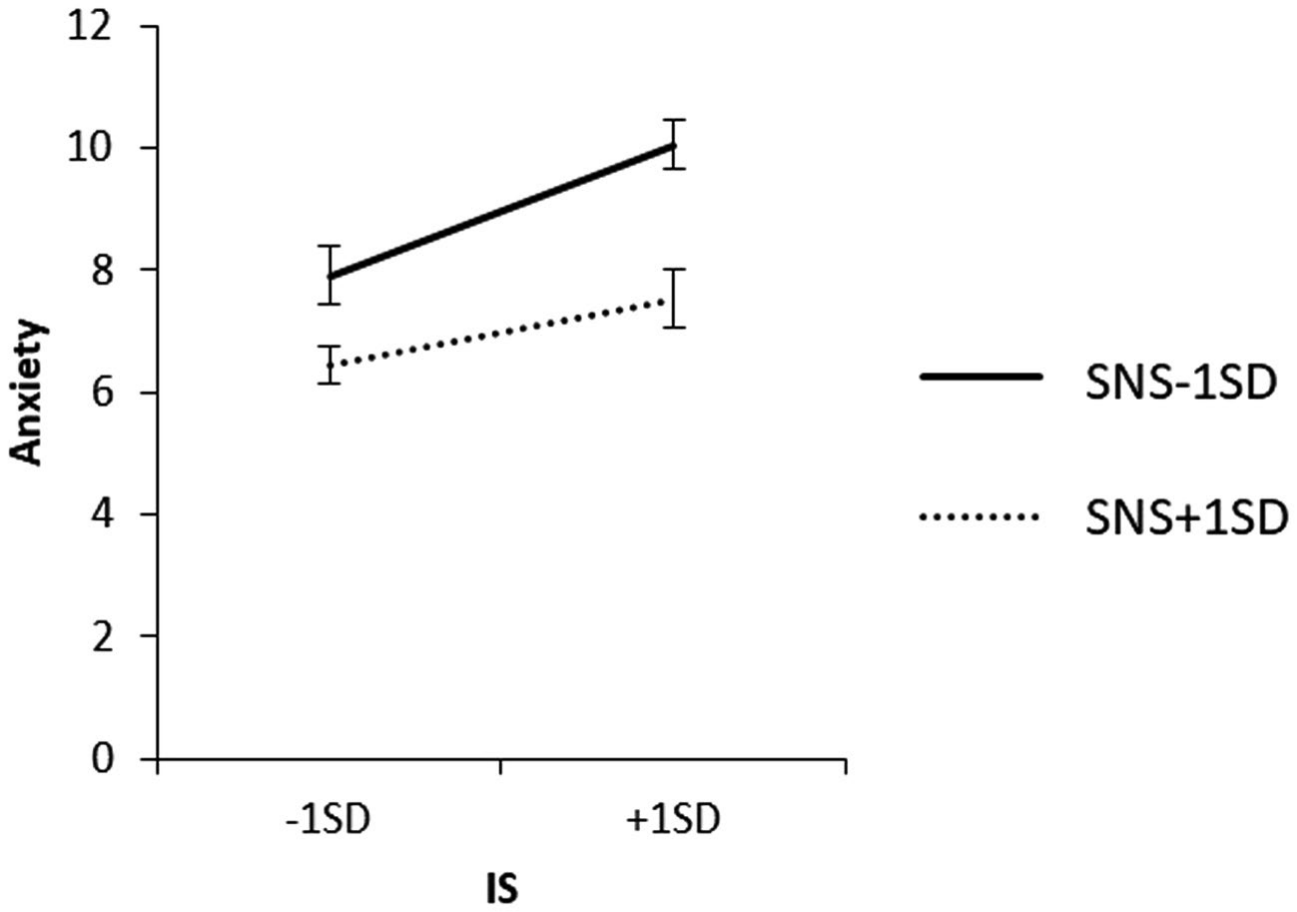
Simple effects analysis of the interaction term of IS × SNS × low household income group for anxiety.

**Figure 5:**
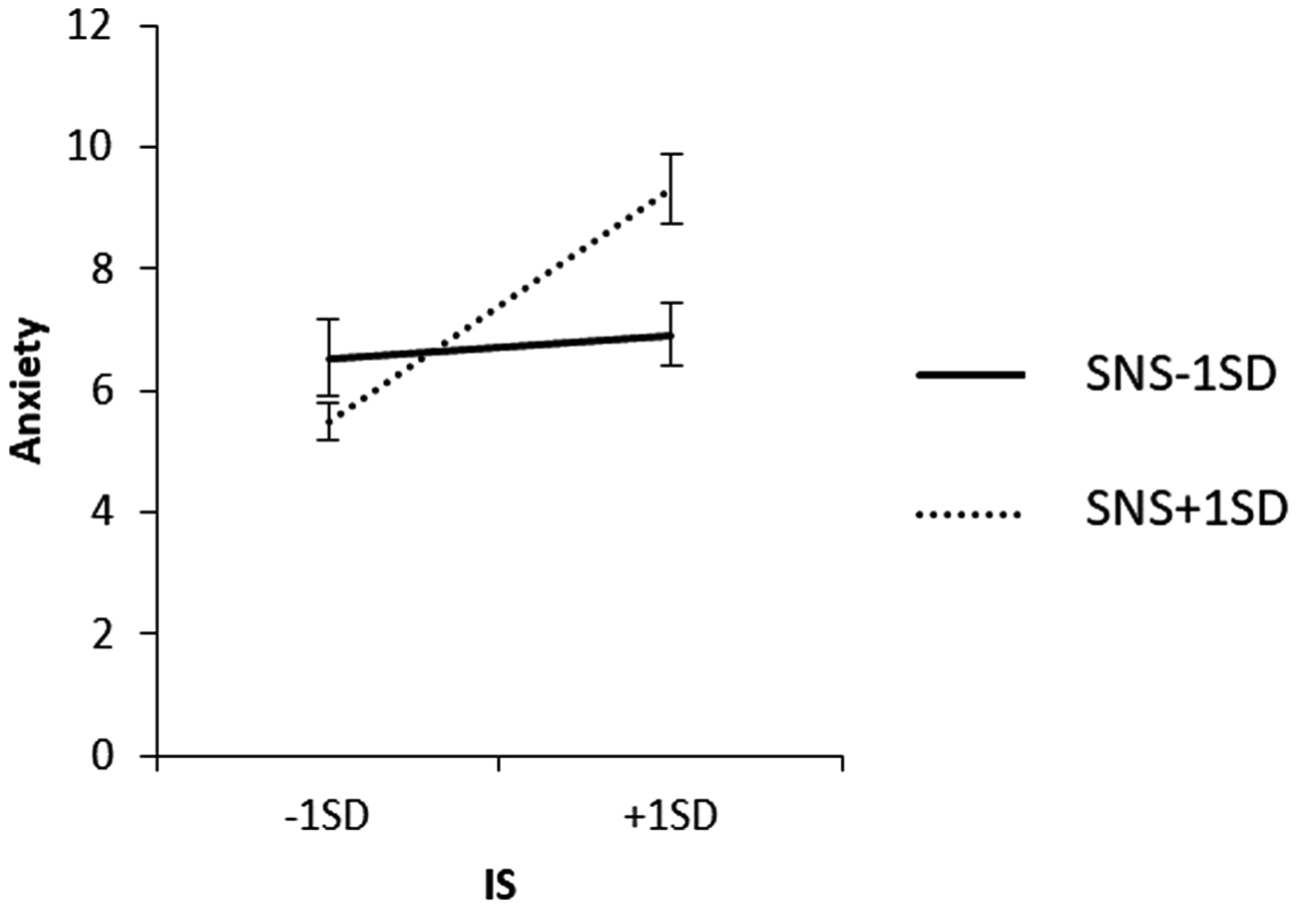
Simple effects analysis of the interaction term of IS × SNS × high household income group for anxiety.

To examine the content of the significant interaction, we used a simple slope analysis to examine the effect of household income on each value of the SNS mean ± 1 SD, and the effect of IS on each value of the SNS mean ± 1 SD for each household income group. The results are shown in Figures 3–5.

As observed from the mean + 1 SD of SNS, household income did not have a significant effect on anxiety when SNS was high (*β* = 0.06, ns). When SNS was high, IS in the low-income group did not have a significant effect on anxiety (*β* = 0.16, ns), and IS in the high-income group did not have a significant effect on anxiety (*β* = 0.16, ns). IS had a significant positive effect on anxiety (*β* = 0.57, *p* < 0.01).

When SNS was low, the low-household income group had a significant positive effect on anxiety (*β* = 0.32, *p* < 0.01), and the high-household income group had no significant effect on IS (*β* = 0.06, ns).

The procedure for the hierarchical multiple regression analysis with stress as the objective variable was as follows: in Step 1, the subscales of the SAIS, IS, SNS, and household income were entered into the regression equation. In Step 2, we added the interaction terms of IS and SNS, IS and household income, and SNS and household income to the variables in Step 1. In Step 3, the interaction terms of IS, SNS, and household income were added to the variables in Steps 1 and 2.

The results of the hierarchical multiple regression analysis with stress as the objective variable are shown in Table 7, where the interaction terms of IS, SNS, and household income were positively associated with stress, while SNS and household income were negatively associated with stress. The increase in *R*^2^ from Steps 1 to 2 and from Steps 2 to 3 was 0.01 (*F* = 3.26, *p* < 0.01) and 0.01 (*F* = 6.07, *p* < 0.01), respectively.

**Table 7:** Hierarchical multiple regression analysis with stress as the objective variable.

	*R* ^2^	Δ*R*^2^	Adj.*R*^2^	*β*
Step 1	0.18^**^		0.17	
IS				0.30^**^
SNS				−0.18^**^
Household income				−0.15^**^
Step2	0.19^**^	0.02^*^	0.18	
IS				0.31^**^
SNS				−0.15^**^
Household income				−0.22^**^
IS × SNS				−0.02
IS × Household income				−0.07
SNS × Household income				−0.17^**^
Step3	0.20^**^	0.01^*^	0.19	
IS				0.30^**^
SNS				−0.13^**^
Household income				−0.19^**^
IS × SNS				0.08^*^
IS × Household income				0.06
SNS × Household income				0.20^**^
IS × SNS × Household income				0.13^*^

IS, Insecure strivings; SNS, Secure non-strivings.

* *p* < 0.05;

** *p* < 0.01.

**Figure 6:**
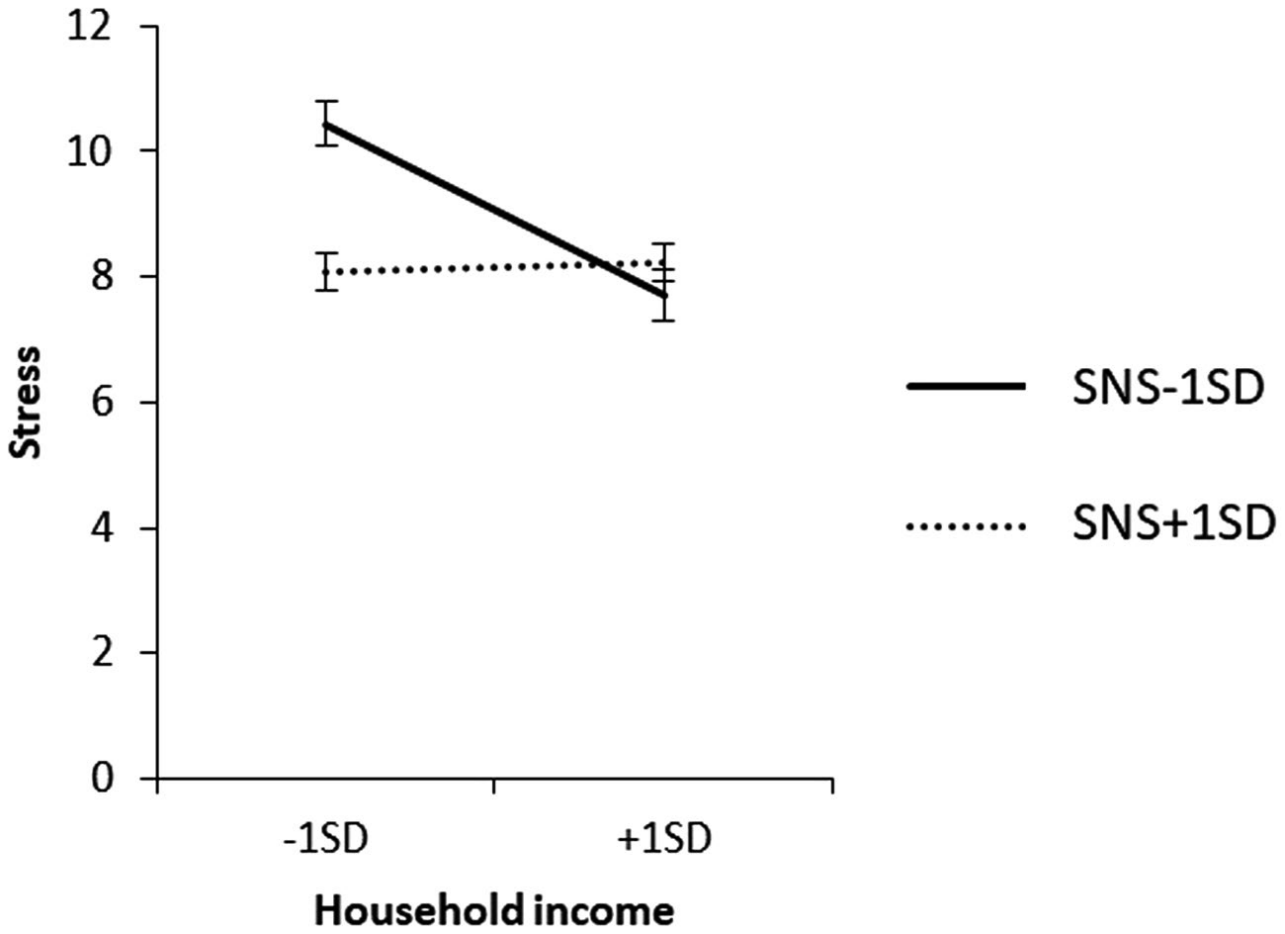
Simple effects analysis of the interaction term SNS × household income for stress.

**Figure 7:**
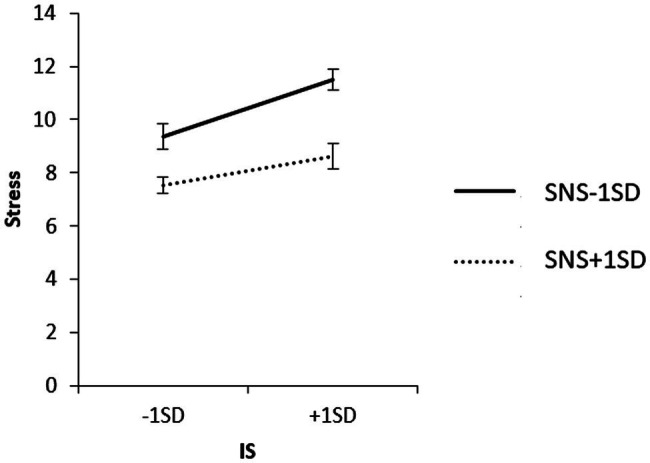
Simple effects analysis of the interaction term of IS × SNS × low household income group for stress.

**Figure 8:**
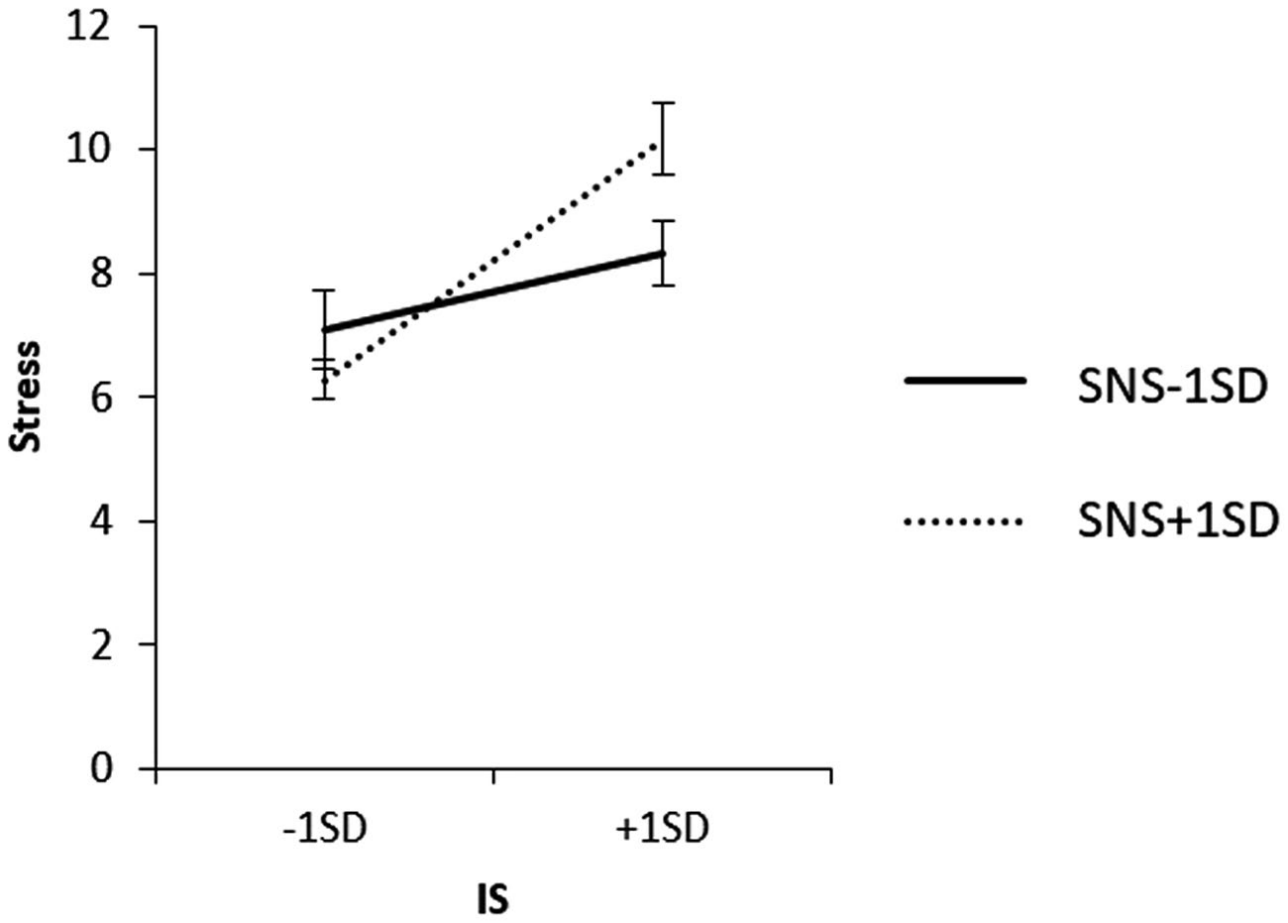
Simple effects analysis of the interaction term of IS × SNS × high household income group for stress.

To examine the content of the significant interaction, we used a simple slope analysis to examine the effect of household income on each value of the SNS mean ± 1 SD, and the effect of IS on each value of the SNS mean ± 1 SD for each household income group. The results are shown in Figures 6–8.

The results of the simple slope analysis are shown in Figures 6–8. First, the results of the mean value of SNS + 1 SD showed that household income did not have a significant effect on stress when SNS was high (*β* = 0.02, ns). When SNS was high, the IS of the high-household income group had a significant positive effect on stress (*β* = 0.56, *p* < 0.01).

When SNS was low, the low-household income group had a significant positive effect on stress (*β* = 0.31, *p* < 0.01), and the IS in the high-household income group had no significant effect on stress (*β* = 0.18, ns).

## Discussion

The purpose of this study was to examine (1) the relationship between striving to avoid inferiority and household income, mental health, and well-being and (2) whether striving to avoid inferiority is a factor that moderates the relationship between household income and mental health or well-being.

The results of correlation analysis showed that IS correlated with mental health and SNS correlated with well-being and mental health. These results consistent with previous studies (Bellew et al., 2006; Gilbert et al., 2007; Walton et al., 2020; To et al., 2021). Such injurious role of IS and protective role of SNS are shown as direct effects in the moderator analyses too. These findings strengthen the evidence that IS works as risk factor on mental health but SNS works as protective one. SNS was associated with well-being, but IS was not associated with well-being. This may be due to the interaction of IS with other factors such as SNS and income, as well as the fact that well-being and IS are not correlated. Besides, correlations between household income and mental health or well-being were small. These results may indicate that the relationships between household income and mental health or well-being are not linear as pointed out by Kahneman and Deaton (Kahneman and Deaton, 2010).

Next, we conducted hierarchical multiple regression analyses and results showed the following findings. Higher SNS and household income consistently related to better mental health and well-being but IS related to worse mental health similar to result of correlation analyses. Furthermore, individuals with either SNS or household income can maintain mental health and well-being. SNS is a sense of acceptance of oneself by others regardless success or failure, which can provide individuals with social safeness. This is consistent with an old report suggesting that social supportive network relates to well-being and ill-being (Headey et al., 1984), and suggesting that social connectedness is an essential part for people. About household income, previous studies pointed out that socioeconomic status relates to lower mental health (Pinquart and Sörensen, 2000; Lorant et al., 2003; Wang et al., 2010), and we can suggest that sufficient economic state is also an essential part.

Interestingly, the results on anxiety and stress suggested that when all of IS, SNS, and household income were higher, anxiety and stress also became higher. This means that protective roles of SNS and household income were weakened if higher IS coexists. Individuals with higher SNS, household income and IS seemed to have both of assets and reliable interpersonal relationship which preventing from depression and having satisfaction with life. However, if they had higher IS, they might be frightened to lose money or relationship and strive to avoid inferiority because they are haves. For example, if they have safe relationship with family including dependents, they need to earn money in competitive relationship at workplace because of the importance of dependents. Although it is difficult to determine why such interaction caused, such interaction of positive and negative factors has long been discussed (Headey et al., 1984), and balancing both of the aspects may contribute to better states (Trompetter et al., 2017).

For the above results, some suggestions can be made. First, the results suggested that financial support for low-income individuals was essential in improving mental health as suggested in previous research (Sareen et al., 2011). In the Finnish basic income experiment, respondents who received basic income reported less depression, sadness, and loneliness (Kela, 2020). Next, mental health can be improved by increasing SNS as previous studies have shown that a sense of acceptance or social support are related to mental health (Sugiyama, 2004; Harandi et al., 2017; Liu et al., 2021). Third, not only approaching income and SNS, it is also important to ease IS which can damage mental health even though individuals have higher income and social connectedness. IS is regarded as a part of competitive mentalities (Gilbert et al., 2009), which can be shifted to caring mentalities *via* psychotherapy such as compassion focused therapy (Gilbert, 2019). Therefore, developing compassion-based interventions for individuals with higher IS regardless of income or interpersonal satisfaction would be needed. At the social level, the liberal attitude of stirring up competition may also need to be reviewed.

Although this study presented insightful findings, there are several limitations, and further studies are required. The first one is the target population of the study. The survey was conducted only in Japan. Therefore, it is necessary to conduct a similar survey outside Japan in the future and examine the similarities and differences. The second is the sample size. In previous studies investigated income, sample sizes of over 3,000 were used. Therefore, it is necessary to conduct a large-scale survey in the future to validate the results of this study. The third is the method of measuring income. The measurement of income was based on self-application; therefore, there is no objective information, and there is a possibility of false reporting. The fifth is that we did not examine gender differences. It is assumed that gender can impact on social role, job or income (Navarro and Salverda, 2019), and should be revealed in the future. Finally, since this study was conducted during a pandemic, it is suggested that IS and SNS were affected by the pandemic to an extent; therefore, it is desirable to conduct another survey in a non-pandemic situation.

## Data Availability Statement

The raw data supporting the conclusions of this article will be made available by the authors, without undue reservation.

## Ethics Statement

The studies involving human participants were reviewed and approved by the Research Ethics Committee of Mejiro University in the Humanities and Social Sciences. Written informed consent for participation was not required for this study in accordance with the national legislation and the institutional requirements.

## Author Contributions

AN and KA contributed to conception and design of the study and wrote the first draft of the manuscript. KA and YK translated SAIS into Japanese. KA conducted survey. AN performed the statistical analysis. YK wrote sections of the manuscript. All authors contributed to the article and approved the submitted version.

## Funding

This work was supported by JSPS KAKENHI grant number 19H01764.

## Conflict of Interest

The authors declare that the research was conducted in the absence of any commercial or financial relationships that could be construed as a potential conflict of interest.

## Publisher’s Note

All claims expressed in this article are solely those of the authors and do not necessarily represent those of their affiliated organizations, or those of the publisher, the editors and the reviewers. Any product that may be evaluated in this article, or claim that may be made by its manufacturer, is not guaranteed or endorsed by the publisher.

## Abbreviations

IS: Insecure striving
SNS: Secure non-striving
SWLS: Satisfaction with life scale
SAIS: Striving to avoid inferiority scale
DASS: Depression anxiety stress scale
