# Corrigendum: Moderating effects of striving to avoid inferiority on income and mental health

**DOI:** 10.3389/fpsyg.2022.992619

**Published:** 2022-09-26

**Authors:** Asa Nagae, Kenichi Asano, Yasuhiro Kotera

**Affiliations:** ^1^Graduate School of Psychology, Mejiro University, Tokyo, Japan; ^2^National Center for Cognitive Behavior Therapy and Research, National Center of Neurology and Psychiatry, Tokyo, Japan; ^3^School of Health Sciences, University of Nottingham, Nottingham, United Kingdom

**Keywords:** inferiority, mental health—related quality of life, well-being, income, social safeness, social comparison

In the published article, there was an error in the affiliations as published. Affiliation 1 was written as “National Center for Cognitive Behavior Therapy and Research, National Center of Neurology and Psychiatry, Tokyo, Japan” but should be “Graduate School of Psychology, Mejiro University, Tokyo, Japan.” Affiliation 2 was written as “Department of Psychology, Mejiro University, Tokyo, Japan” but should be “National Center for Cognitive Behavior Therapy and Research, National Center of Neurology and Psychiatry, Tokyo, Japan.”

In addition, there was an error in the affiliations for Asa Nagae. They were originally written as “Asa Nagae1” but should be “Asa Nagae^1, 2^.”

The authors apologize for this error and state that this does not change the scientific conclusions of the article in any way. The original article has been updated.

## Publisher's note

All claims expressed in this article are solely those of the authors and do not necessarily represent those of their affiliated organizations, or those of the publisher, the editors and the reviewers. Any product that may be evaluated in this article, or claim that may be made by its manufacturer, is not guaranteed or endorsed by the publisher

